# Value of Dual-Energy Dual-Layer CT After Mechanical Recanalization for the Quantification of Ischemic Brain Edema

**DOI:** 10.3389/fneur.2021.668030

**Published:** 2021-07-19

**Authors:** Paul Steffen, Friederike Austein, Thomas Lindner, Lukas Meyer, Matthias Bechstein, Johanna Rümenapp, Tristan Klintz, Olav Jansen, Susanne Gellißen, Uta Hanning, Jens Fiehler, Gabriel Broocks

**Affiliations:** ^1^Department of Diagnostic and Interventional Neuroradiology, University Medical Center Hamburg-Eppendorf, Hamburg, Germany; ^2^Department of Radiology and Neuroradiology, University Hospital Schleswig-Holstein, Kiel, Germany

**Keywords:** net water uptake, mechanical recanalization, dual-energy computed tomography, virtual non-contrast image, brain edema, ischemia, acute stroke

## Abstract

**Background and Purpose:** Ischemic brain edema can be measured in computed tomography (CT) using quantitative net water uptake (NWU), a recently established imaging biomarker. NWU determined in follow-up CT after mechanical thrombectomy (MT) has shown to be a strong predictor of functional outcome. However, disruption of the blood–brain barrier after MT may also lead to contrast staining, increasing the density on CT scans, and hence, directly impairing measurements of NWU. The purpose of this study was to determine whether dual-energy dual-layer CT (DDCT) after MT can improve the quantification of NWU by measuring NWU in conventional polychromatic CT images (CP-I) and virtual non-contrast images (VNC-I). We hypothesized that VNC-based NWU (vNWU) differs from NWU in conventional CT (cNWU).

**Methods:** Ten patients with middle cerebral artery occlusion who received a DDCT follow-up scan after MT were included. NWU was quantified in conventional and VNC images as previously published and was compared using paired sample *t*-tests.

**Results:** The mean cNWU was 3.3% (95%CI: 0–0.41%), and vNWU was 11% (95%CI: 1.3–23.4), which was not statistically different (*p* = 0.09). Two patients showed significant differences between cNWU and vNWU (Δ = 24% and Δ = 36%), while the agreement of cNWU/vNWU in 8/10 patients was high (difference 2.3%, *p* = 0.23).

**Conclusion:** NWU may be quantified precisely on conventional CT images, as the underestimation of ischemic edema due to contrast staining was low. However, a proportion of patients after MT might show significant contrast leakage resulting in edema underestimation. Further research is needed to validate these findings and investigate clinical implications.

## Introduction

Randomized control trials demonstrated that mechanical thrombectomy (MT) of anterior large vessel occlusion (LVO) in acute stroke patients improves the clinical outcome compared to standard therapy (1). Yet, the clinical outcome, even after successful recanalization varies widely (2), and is subject of ongoing investigations. Different parameters influence the outcome, for example, the time from clinical onset to reperfusion, patient age, the National Institutes of Health Stroke Scale (NIHSS) at admission, the Alberta stroke program early computed tomography (ASPECT) score at admission, and the baseline functional status (3).

Recently, it has been observed that the degree of edema formation in early follow-up imaging captured by net water uptake (NWU), a quantitative imaging biomarker, is an indicator of the response to MT (4). NWU is an accurate predictor of functional outcome and outperforms clinical variables, for example, age, NIHSS, and/or ASPECTS (4). These results are in accordance with other studies describing a strong correlation between NWU quantified in CT and the final infarct volume (5, 6).

Cerebral edema is the pathophysiological response of ischemic brain tissue undergoing infarction, which is evident in CT by means of progressive tissue hypoattenuation (7). Reperfusion after LVO has been associated with increased edema formation caused by microvascular damage probably resulting in an extension of the initial infarct area (8), but recently, it has been observed that MT might reduce cerebral edema (9, 10) and is associated with a lower risk for progressive edema compared to intravenous thrombolysis (11).

Disruption of the blood–brain barrier after MT may not only lead to edema but also to hemorrhage and/or contrast staining (12), increasing the density on CT scans, and hence, directly impairing measurements of brain edema on conventional polychromatic CT images (CP-I). Lesion hyperattenuations are common findings after LVO and are described in up to 84% of patients directly after MT, and ~20% after 24 h, which could result in an underestimation of brain edema on conventional polychromatic images (CP-I) (13). Follow-up CT is routinely performed after MT, in particular to rule out hemorrhage (14), but differentiation between tissues densities of similar Hounsfield units, for example, blood and contrast medium remains difficult in this modality.

Recently, dual-energy imaging methods have become increasingly popular in research and clinical practice because of their capability to discriminate between tissues of similar X-ray attenuation but different atomic numbers (15–19). Dual-energy CT technology is based upon the light-matter interaction and different absorption characteristics of specific substances. Iodine for instance increases X-ray absorption at 32.2 keV (16, 20). Different technical methods are available to capture these characteristic absorption profiles of substances and generate dual-energy CT datasets, which can be used to calculate virtual non-contrast images (VNC-I).

Considering the clinical relevance of cerebral edema as an indicator for malignant infarction as well as for outcome prediction, we aim to investigate the potential underestimation of edema on conventional CT images due to contrast staining by using DDCT scans for VNC-based NWU quantification. We hypothesized that NWU quantification in follow-up imaging after MT differs significantly between conventional polychromatic and VNC images.

## Materials and Methods

### Study Population

The data that support the results of this study are available from the corresponding author, upon reasonable request. The local ethics committee approved the study (Ethics Committee of the Medical Faculty of the Christian-Albrechts-University, Kiel; Number D 567/18) and waived the requirement to obtain informed consent. We retrospectively analyzed the data of 10 consecutive patients referred to our hospital between September 2019 and January 2020 who received DDCT scans of the cranium after MT of a LVO of the anterior circulation within the standard clinical protocol. MT was performed according to the ESO/ESMINT-Guidelines (21). Data was anonymized and analyzed, retrospectively.

### Image Acquisition and Reconstruction

Image acquisition was performed on a dual-energy dual-layer CT (IQon spectral CT, Philips Healthcare, USA) with 120 kVp and 230 mAs. IntelliSpace (Version 11.1, Philips Healthcare, USA) was used as post-processing software. The VNC-I and the CP-I were generated from spectral based datasets and reformatted with 5-mm slice thickness. The CP-I were reconstructed using iterative model-based reconstructions (level 1, filter UB), whereas the VNC-I were generated using a spectral reconstruction mode (level 2).

### Image Analysis

The data were analyzed using IntelliSpace (Version 11.1, Philips Healthcare, USA). The rater was blinded for all patient-related data and clinical information. A standardized procedure to quantify the proportion of ischemic edema due to NWU was used as previously published (5, 22). In summary, a region of interest (ROI) was placed for density measurements according to the extent of ischemic hypoattenuation identified on conventional images with hindsight knowledge of VNC-I, and the core lesion in CT perfusion imaging was mirrored to the unaffected contralateral brain hemisphere. ROI histograms were sampled between 20 and 80 Hounsfield units to exclude voxels belonging to calcifications or cerebrospinal fluid. Both measurements were then used to calculate the proportion of edema within the lesion, obtaining NWU of CP-I (cNWU) (23, 24). This procedure was subsequently repeated on VNC-I to calculate VNC-based NWU (vNWU).

### Statistical Analysis

Data is reported using standard descriptive statistics. All statistics were calculated using MedCalc (version 11.5.1.0, Mariakerke, Belgium). *P*-values < 0.05 were considered statistically significant. cNWU and vNWU were compared using paired sample *t*-tests with means and 95% confidence intervals. The difference of both measurements was compared for every patient (ΔNWU = vNWU – cNWU). Bland-Altman plots were used to illustrate measurements of vNWU and cNWU for every patient (**Figure 2**).

## Results

In this pilot study, 10 consecutive patients (five female and five male) were included with mean age of 81 years (range: 53–99). Within this group, patients had been examined 12h (± 6h) after MT (median 9.5h). The pattern of vessel occlusion was nine M1-occlusions and one proximal M2-occlusion. Post-interventional TICI score was 2b-3 in nine cases and 2a in 1 case. The median ASPECTS was 8 (range: 4–10), and the mean core lesion volume defined by regional cerebral blood flow <30% was 11.5 ml (range: 0–57 ml). The median NIHSS was 14 (interquartile range: 6–18).

**Figure 1:**
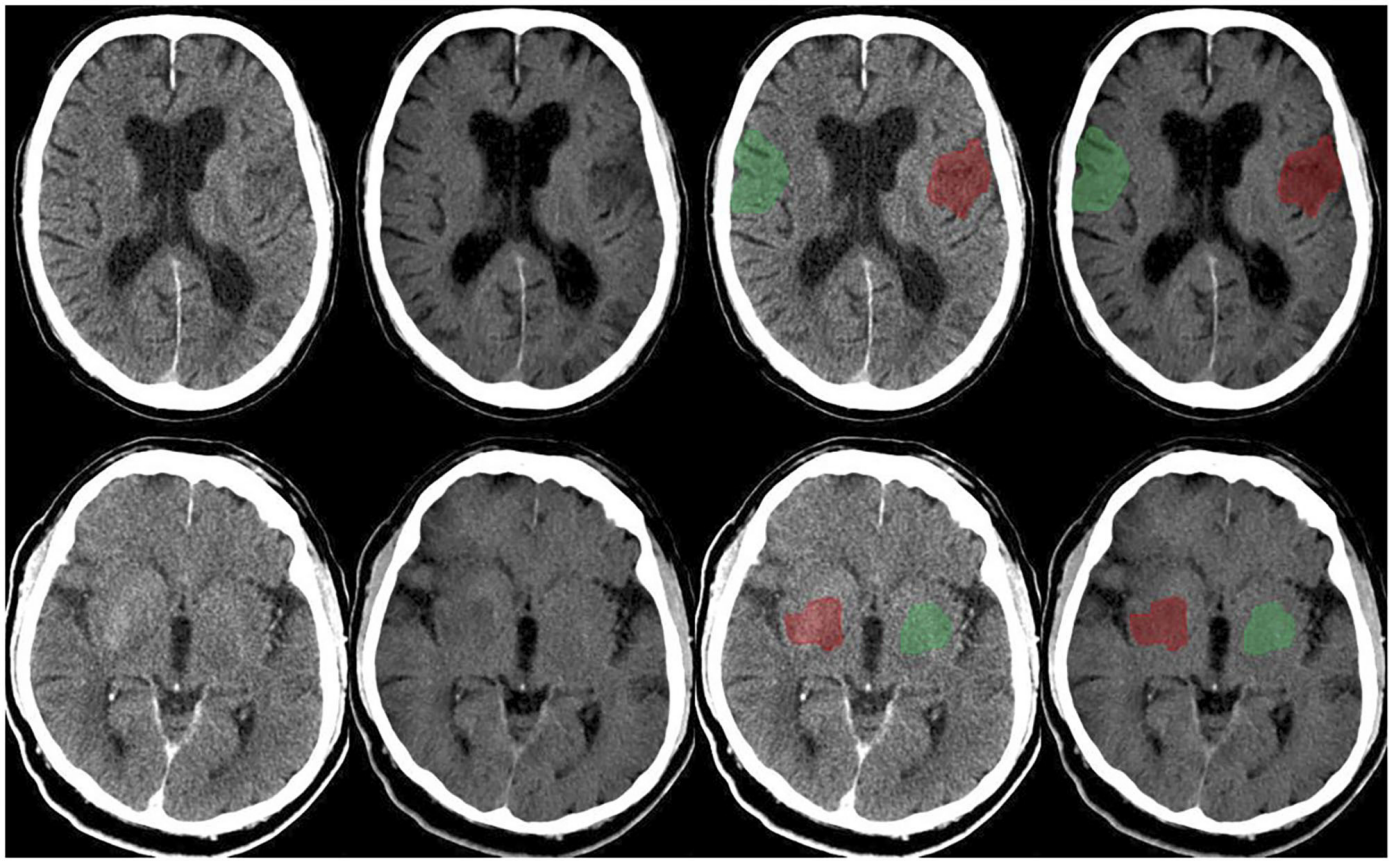
Upper row: Follow-up dual-layer dual-energy CT within 12 h in a 70-year-old man with left MCA occlusion (initial NHISS 7; ASPECTS 8) after thrombectomy (TICI 3), time onset to reperfusion 184 min. Conventional polychromatic image (first and third from the left), VNC image (second and fourth from the left). Example of ischemic ROI placement (red) and contralateral normal ROI placement (green). Lower row: Follow-up dual-layer dual-energy CT within 12 h in an 89-year-old man with right MCA occlusion (initial NHISS 17; ASPECTS 8) after thrombectomy (TICI 3), time onset to reperfusion 154 min. Conventional polychromatic image (first and third from the left), VNC image (second and fourth from the left). Example of ischemic ROI placement (red) and contralateral normal ROI placement (green).

Figure 1 demonstrates the CP-I and VNC-I, which were used for further image analysis. Overall, the mean NWU was 3.3% (95%CI: 0–0.41%) in cNWU and 11% (95%CI: 1.3–23.4%) in vNWU. The mean difference between cNWU and vNWU was 7.7%, which was not statistically different (*p* = 0.09).

**Figure 2:**
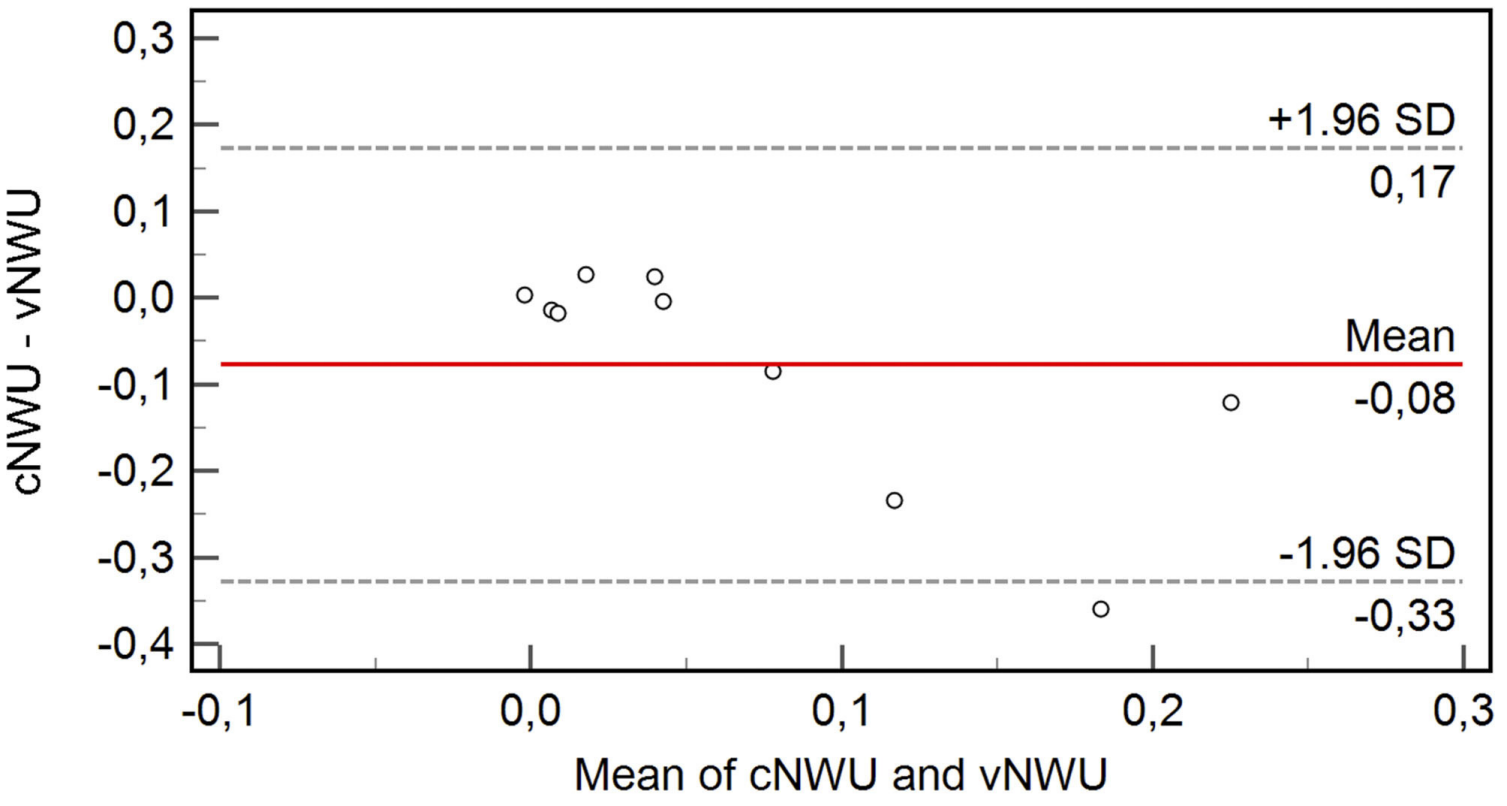
Bland-Altman Plot showing the variable difference of cNWU and vNWU in percent. The mean difference between both methods was −0.08 with only one patient outside ±1.96 standard deviations.

Eight out of 10 patients showed a high agreement of NWU with a mean difference of only 2.3% between cNWU and vNWU (*p* = 0.23). Two out of 10 patients were significant outliers with high differences in NWU between cNWU and vNWU of 24 and 36%, respectively. For other aspects, these two patients were in range of the cohort. Age was 70 (86) years, ASPECTS was 8 (8), final TICI was 3 (3), core size lesion 0 (28) ml, and time from MT to follow-up CT scan was 7 h (10 h). The Bland-Altman Plot (Figure 2) shows the exact differences in NWU for each patient in percent.

## Discussion

This is the first study to investigate VNC-I for the use of NWU quantification, targeting a potential underestimation of cerebral edema in conventional CT images due to contrast staining in stroke lesions. This was done by comparing NWU in CP-I and VNC-I from DDCT scans, using a previously published method (6). The benefit of VNC-I to visually improve the detection of cerebral edema in acute stroke lesions was shown (25, 26), but measuring the impact of VNC-I on NWU quantification has not been investigated before.

The main finding of this pilot study was that cNWU and vNWU did not show a significant difference in quantified NWU (*p* = 0.09). By trend, vNWU had a slightly higher NWU compared to cNWU (11 vs. 3.3.%), which was mainly caused by two outliers in our study group showing significant differences in NWU of 24 and 36%, respectively (Figure 2). The agreement of the other eight patients was high. This implies the possibility of a small subgroup of patients, which might show a stronger than average hyperattenuation in CP-I and thus an underestimation of true cerebral edema in cNWU. The underlying cause remains uncertain but possible cofounders influencing contrast staining in stroke lesions are: kidney function, diagnostic multimodal CT examinations before MT, and the recanalization procedure itself, resulting in different contrast agent preloads. Another already described cofounder to impact cNWU measurements is the time at which the follow-up CT is taken. Lesion hyperattenuations are decreasing in time with up to 84% patients having hyperattenuated lesions directly after MT but only 20% of patients after 24 h (6, 13). Detailed data describing the dynamics of hyperattenuations in acute and subacute stroke lesions are still lacking, and further investigations are needed to determine the temporal relationships.

Our results support previous studies describing NWU in acute stroke lesions as a reliable quantitative imaging biomarker to measure edema in acute stroke lesions (9, 23, 24, 27–29). NWU hereby demonstrates a more accurate quantification of cerebral edema than other methods, such as midline shift measurements, which may significantly depend on age and volume of cerebrospinal fluid (30). This is in accordance with studies showing that NWU is a good predictor of functional outcome and does so more accurately than clinical variables (e.g., NIHSS) or final infarct volume (4–6), while other parameters like ASPECS show low interrater agreement reliability (31). Thus, NWU could serve as an interesting biomarker and imaging end point in stroke trials.

We hypothesized that the densitometric assessment of NWU could be impaired significantly by, even visually inapparent, iodine contrast staining on CP-I after MT, leading to an underestimation of cerebral edema. But the agreement between cNWU and vNWU was high in eight out of 10 patients showing a mean difference of only 2.3%, confirming cNWU and vNWU as a reliable imaging source to quantify NWU.

To our best knowledge, this is the first study to investigate whether residual contrast enhancement affects quantification of NWU in CP-I. As our results demonstrate: contrast staining after MT does not alter the measurement of NWU on CP-I significantly. However, a certain proportion of patients showed a significant difference in NWU between cNWU and vNWU, suggestive to interindividual factors influencing contrast staining.

Designed as a pilot and feasibility study, the number of included patients was small, resulting in low power. Yet, our results confirm previous studies that NWU can be determined reliably on CP-I as well as VNC-I and stimulate future research regarding the potential benefit of VNC-based NWU quantification.

In conclusion, this is the first study to investigate NWU using dual-energy dual-layer CT scans, demonstrating a strong agreement between cNWU and vNWU. Significant differences in cNWU and vNWU are seen in a small subgroup of patients, which should be subject for further research.

## Data Availability Statement

The raw data supporting the conclusions of this article will be made available by the authors, without undue reservation.

## Ethics Statement

The studies involving human participants were reviewed and approved by Ethics Committee of the Medical Faculty of the Christian-Albrechts-University, Kiel; Number D 567/18. Written informed consent for participation was not required for this study in accordance with the national legislation and the institutional requirements.

## Author Contributions

PS, FA, and GB: conceptualization, planning, research, data management, statistical analysis, interpretation of results, and writing. TL: advisor on physical topics. SG: advisor on statistical analysis. JR and TK: help with data aquisition. LM, MB, UH, JF, and OJ: advisor on writing, interpretation of results, and proofreading. All authors contributed to the article and approved the submitted version.

## Conflict of Interest

JF reports grants and personal fees from Acandis, grants and personal fees from Cerenovus, grants and personal fees from Microvention, grants and personal fees from Medtronic, grants and personal fees from Stryker, grants from Route 92, personal fees from Phenox, personal fees from Penumbra, outside the submitted work. OJ reports grants and personal fees from Acandis, grants and personal fees from Cerenovus, grants and personal fees from Microvention, grants and personal fees from Boehringer, personal fees from Bayer, and personal fees from Philips, outside the submitted work. The remaining authors declare that the research was conducted in the absence of any commercial or financial relationships that could be construed as a potential conflict of interest.
